# Tropomyosin-related kinase B mediated signaling contributes to the induction of malignant phenotype of gallbladder cancer

**DOI:** 10.18632/oncotarget.16063

**Published:** 2017-03-10

**Authors:** Makoto Kawamoto, Hideya Onishi, Keigo Ozono, Akio Yamasaki, Akira Imaizumi, Sachiko Kamakura, Kenji Nakano, Yoshinao Oda, Hideki Sumimoto, Masafumi Nakamura

**Affiliations:** ^1^ Department of Cancer Therapy and Research, Graduate School of Medical Sciences, Kyushu University, Fukuoka, Japan; ^2^ Department of Anatomic Pathology, Graduate School of Medical Sciences, Kyushu University, Fukuoka, Japan; ^3^ Shukoukai Inc., Tokyo, Japan; ^4^ Department of Biochemistry, Graduate School of Medical Sciences, Kyushu University, Fukuoka, Japan; ^5^ Innovation Center for Medical Redox Navigation, Kyushu University, Fukuoka, Japan; ^6^ Department of Surgery and Oncology, Graduate School of Medical Sciences, Kyushu University, Fukuoka, Japan

**Keywords:** TrkB, BDNF, gallbladder cancer, biliary tract cancer, HIF-1α

## Abstract

This study aims to demonstrate the clinical and biological significance of Brain derived neurotrophic factor (BDNF)/Tropomyosin-related kinase B (TrkB) signaling in gallbladder cancer (GBC) through a series of *in vitro* and *in vivo* experiments. TrkB expression was detected in 63 (91.3%) out of 69 surgically resected primary GBC specimens by immunohistochemistry. TrkB expression in the invasive front correlated with T factor (p=0.0391) and clinical staging (p=0.0391). Overall survival was lower in patients with high TrkB expression in the invasive front than in those with low TrkB expression (p=0.0363). *In vitro* experiment, we used five TrkB-expressing GBC cell lines with or without K-ras mutation. TrkB-mediated signaling increased proliferation and the invasiveness by inducing epithelial mesenchymal transition, and activating matrix metalloproteinases-2 (MMP-2) and MMP-9. Inhibition of TrkB-mediated signaling also decreased hypoxia-inducible factor-1α, vascular endothelial growth factor A (VEGF-A), VEGF-C, and VEGF-D expression. *In vivo* experiment, inhibition of TrkB-mediated signaling suppressed tumorigenicity and tumor growth in GBC. These findings demonstrate that TrkB-mediated signaling contributes to the induction of malignant phenotypes (proliferation, invasiveness, angiogenesis, lymphangiogenesis, and tumorigenesis) in GBC, and could be a promising therapeutic target regardless of K-ras mutation status.

## INTRODUCTION

Bile tract cancer (BTC) is the sixth leading cause of cancer death in Japan, and has been reported more frequently in Japan compared with other countries [1]. The BTC encompasses diverse diseases, including cancers of the extrahepatic bile ducts, intrahepatic bile ducts, and gallbladder. Of these cancers, gallbladder cancer (GBC) occurs most frequently and has the poorest prognosis [2]. Complete surgical resection is the only potentially curative treatment; however, most GBC cases have developed into locally advanced disease or have metastasized by the time of diagnosis. This is due to the anatomy of gallbladder; the gallbladder has thin wall and is surrounded by rich vascular and lymphatic systems. In inoperable cases, many patients must rely on chemotherapy and radiation therapy, which are not sufficiently effective, and for which the available regimens are still limited. The 5-year survival rate of such patients is estimated to be < 10% [2]. In recent years, effective targeted molecular therapies have been developed for several solid tumor types, but no such treatment is currently available for GBC. Therefore, development of novel molecular therapeutic targets is strongly required for improving outcomes for patients with GBC.

Tropomyosin-related kinase B (TrkB), a member of the neurotrophin receptor family, acts as a receptor for brain-derived neurotrophic factor (BDNF), neurotrophin-4/5, and neurotrophin-3 [3]. Of these ligands, BDNF has the highest affinity for TrkB and evokes strong physiological activity [4]. BDNF/TrkB signaling plays a key role in neuronal proliferation, differentiation, and survival [5]. However, although BDNF/TrkB signaling has been shown to contribute to aggressive phenotypes in some cancers [6–8], no reports have investigated the biological significance of BDNF/TrkB signaling in GBC. In this study, we evaluate the biological significance of BDNF/TrkB signaling in GBC.

## RESULTS

### TrkB expression in the invasive front correlates with invasion depth and poor patient survival in GBC

**Figure 1:**
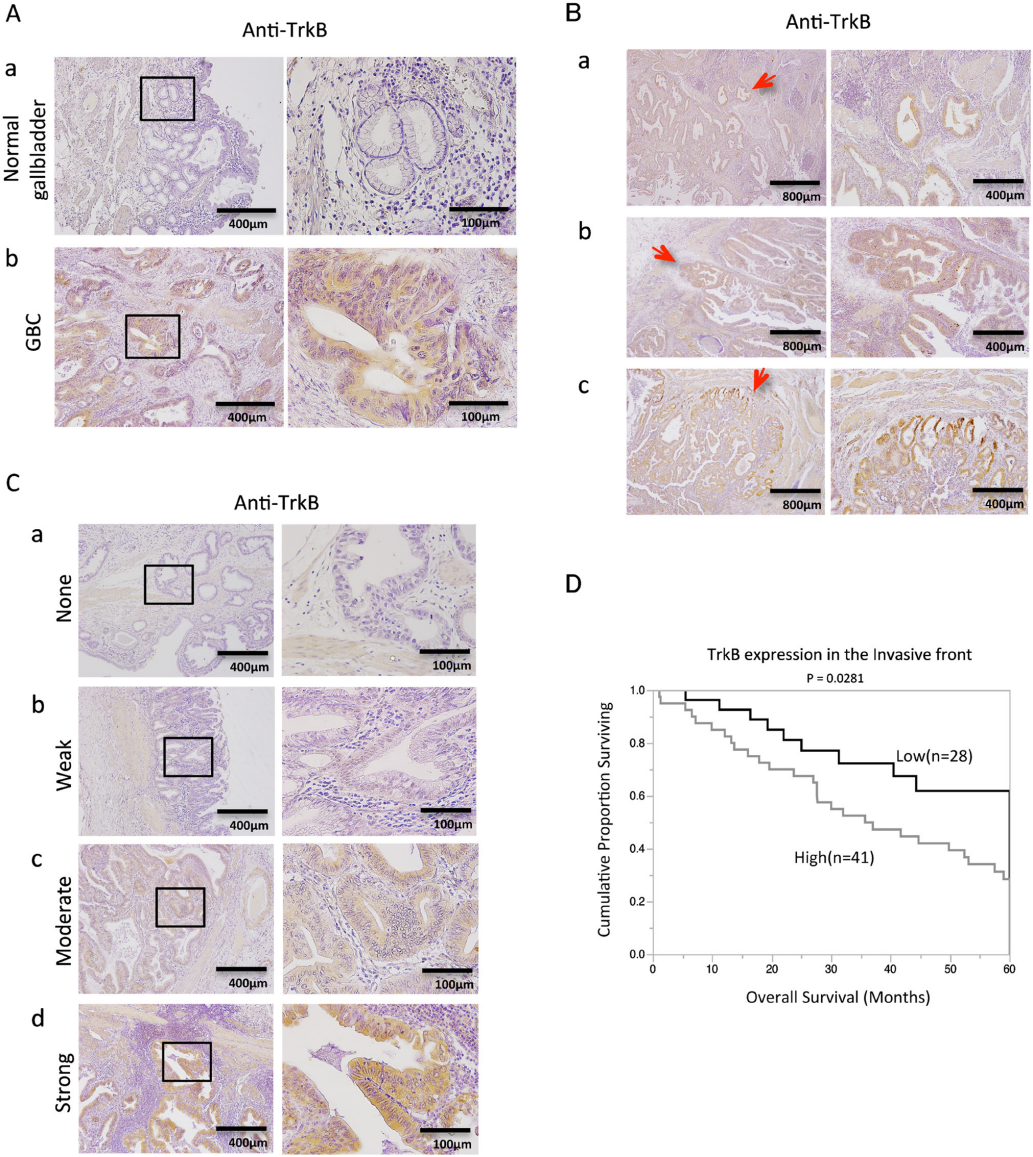
Immunohistochemical analysis of TrkB in primary gallbladder cancer (GBC) **(A)** TrkB expression in normal gallbladder (a), and GBC (b) specimens. Original magnification is 400×(right) and corresponding areas (boxed areas) with lower magnification 100×(left). **(B)** Figures represent invasion front - specific overexpression of TrkB in paraffin-embedded specimens (a-c). Red arrows show the invasive front. Original magnification is 100×(right), 40×(left). **(C)** Representative images of TrkB staining in human GBC specimens: none (a), weak staining (b), moderate staining (c), and strong staining (d). Original magnification is 400×(right) and corresponding areas (boxed areas) with lower magnification 100×(left). **(D)** Kaplan-Meier survival curves of GBC patients according to TrkB expression at the invasive front based on immunohistochemical analysis.

We first investigated TrkB expression by immunohistochemistry in 69 surgically resected human GBC specimens. TrkB expression was detectable in 63 of the 69 GBC specimens (91.3%), whereas no staining was observed in normal gallbladder tissue (Figure 1A). We found that TrkB expression was localized mainly to the cytoplasm of GBC cells. Furthermore, TrkB staining intensity gradually increased from the tumor center to the invasive front in some cases (Figure 1B). We then categorized specimens into 4 groups on the basis of TrkB staining intensity: none, weak, moderate, or strong (Figure 1C). The correlation between TrkB staining intensity and clinicopathological variables was evaluated for the tumor invasive front and the tumor center. Significant positive correlations between TrkB expression in the invasive front and T factor or tumor stage according to UICC classification (P = 0.0391; Table 1) were found by Pearson χ^2^ analysis. Importantly, patients with high TrkB expression in the invasive front had significantly poorer survival than those with low TrkB expression (P = 0.0281; Figure 1D). Taken together, these results suggest that TrkB is overexpressed in most human GBC cases and is involved in cancer invasion depth and poor patient prognosis.

**Table 1:** Clinicopathological findings with primary gallbladder cancer and their correlation to the expression of TrkB intensity in the tumor center and Invasive front

	Total number	TrkB expression inTumor center			TrkB expression inInvasive Front		
		Low	High	P-value	Low	High	P-value
**Gender**				0.3707			0.9083
male	29	15	14		12	17	
female	40	25	15		16	24	
**Age (median=69)**				0.4287			0.6829
<69	30	19	11		13	17	
≧69	39	21	18		15	24	
**T**				0.3847			0.0391*
Tis/1	18	12	6		11	7	
T2/3	51	28	23		17	34	
**Venous invasion**				0.4937			0.5615
Absent	39	24	15		17	22	
Present	30	16	14		11	19	
**Lymphatic invasion**				0.634			0.2036
Absent	38	23	15		18	20	
Present	31	17	14		10	21	
**Lymph node metastasis**				0.068			0.1375
N0	42	28	14		20	22	
N1	27	12	15		8	19	
**Defferentiation**							
G1	44	27	17		15	25	
G2	10	4	6		4	6	
G3	10	5	5		3	7	
G4	0	0	0		0	0	
GX	5	4	1		2	3	
**Stage classification**				0.3847			0.0391*
StageO-I	18	12	6		11	7	
StageII-IIIB	51	28	23		17	34	

Pearsonχ2 test *P<0.05.

### TrkB-mediated signaling is involved in proliferative ability of GBC cell lines

**Figure 2:**
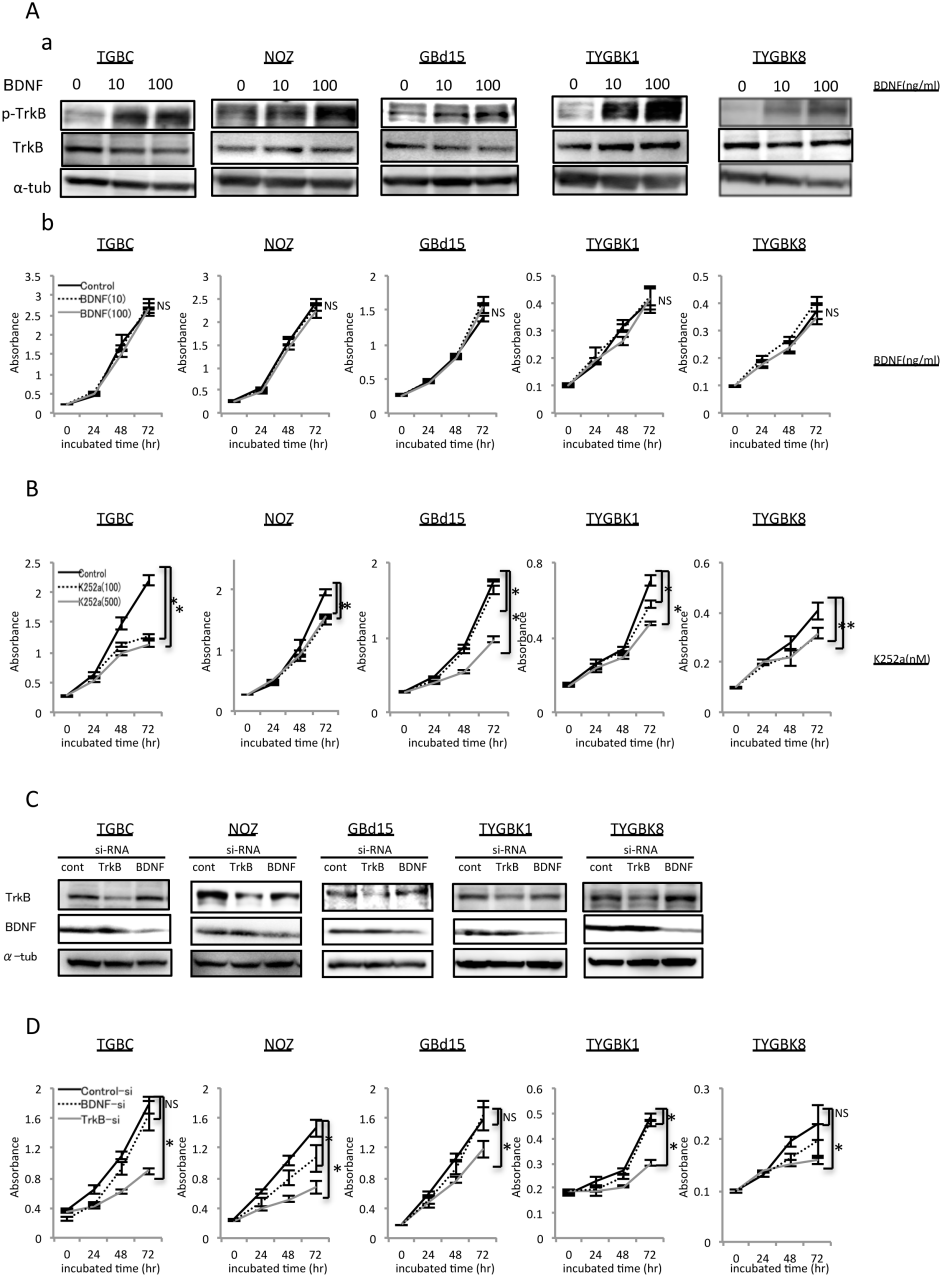
TrkB-mediated signaling is involved in proliferation of GBC cells with expression of endogenous TrkB and BDNF **(A)** Elevated p-TrkB expression by treatment with rhBDNF (at 0, 10, 100 ng/ml) for 18h (a). Proliferation assay of 5 GBC cell lines incubated with rhBDNF (at 0, 10, 100 ng/ml) for 24, 48, or 72 h as indicated (b). **(B)** Proliferation assay of 5 GBC cell lines incubated with K252a (at 0, 100, or 500 nM) for 24, 48, or 72 h. **(C)** Western blot analysis of TrkB and BDNF in 5 GBC cell lines transfected with TrkB siRNA or BDNF siRNA. **(D)** Proliferation assay of 5 GBC cell lines transfected with control siRNA, TrkB siRNA, or BDNF siRNA for 24, 48, or 72 h. *; P<0.05. Bar; SD. NS; not significant.

We hypothesized that BDNF/TrkB signaling may contribute to induction of the malignant potential of GBC. We therefore examined BDNF and TrkB expression in 5 GBC cell lines: TGBC2TKB, NOZ, GBd15, TYGBK-1, TYGBK-8. TrkB and BDNF expression were found in all 5 GBC cell lines (Supplementary Figure 1). On the basis of this result, we used these cell lines to evaluate the impact of BDNF/TrkB signaling activation and inhibition on GBC malignancy *in vitro*. We first examined the role of BDNF/TrkB signaling on GBC proliferation using recombinant human BDNF (rhBDNF) and the Trk tyrosine kinase inhibitor K252a. The phosphorylation of TrkB in GBC cells was observed, as shown in Figure 2A-a. Addition of rhBDNF did not affect the proliferation of any of the cell lines (Figure 2A-b). Conversely, K252a treatment significantly decreased proliferation in all of the cell lines (Figure 2B). To verify a direct link between BDNF/TrkB signaling and proliferation in GBC, we next carried out inhibition experiments using GBC cell lines transfected with TrkB siRNA or BDNF siRNA. Western blotting showed that siRNA knockdown significantly inhibited TrkB and BDNF expression (Figure 2C, Supplementary Figure 2). TrkB siRNA transfection significantly inhibited proliferation in all of the cell lines, whereas BDNF siRNA transfection did not induce a significant difference in proliferation in TGBC2TKB, GBd15, or TYGBK-8 cells (Figure 2D). The above results indicate that the proliferative potential of GBC may be controlled through TrkB-mediated signaling.

### BDNF/TrkB signaling contributes to invasive phenotype in GBC cell lines

**Figure 3:**
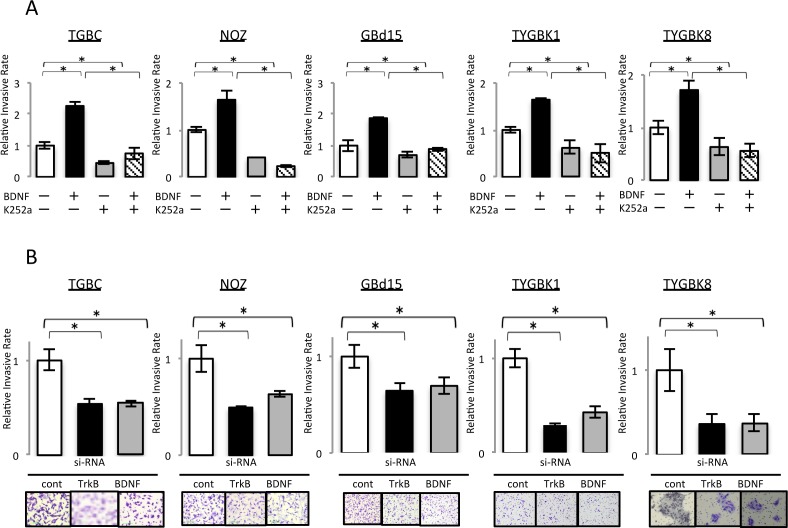
BDNF/TrkB signaling contributes to the invasive phenotype of GBC cells with expression of endogenous TrkB and BDNF **(A)** Invasion assay of 5 GBC cell lines incubated for 18 h with rhBDNF (100 ng/ml) with or without K252a (500 nM) preincubation. **(B)** Invasion assay of 5 GBC cell lines transfected for 18 h with control siRNA, TrkB siRNA, or BDNF siRNA. *; P<0.05. Bar; SD. Original magnification is 100×.

We next analyzed the effect of BDNF/TrkB signaling on GBC invasiveness. Addition of rhBDNF significantly increased Matrigel invasion by all of the cell lines tested in a dose-dependent manner (Supplementary Figure 3A). K252a treatment significantly decreased the invasion by these cell lines (Supplementary Figure 3B). Additionally, the rhBDNF-invoked enhancement of cell invasion was abrogated by K252a (Figure 3A). Finally, TrkB siRNA and BDNF siRNA transfection significantly inhibited the invasiveness of GBC (Figure 3B). Thus, we conclude that BDNF/TrkB signaling contributes to invasive phenotype in GBC.

### BDNF/TrkB signaling-induced invasion is mediated through epithelial–mesenchymal transition (EMT)

**Figure 4:**
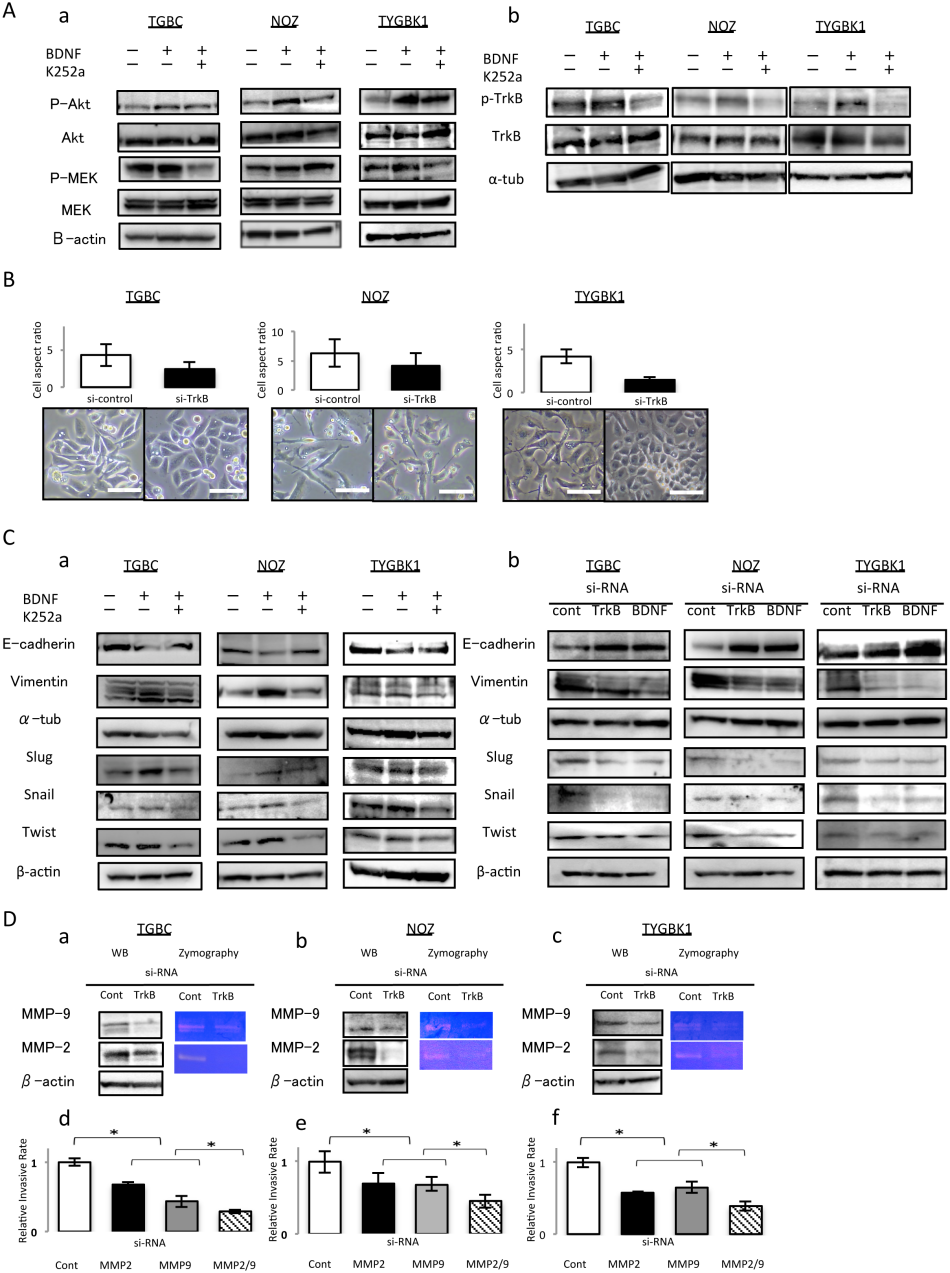
TrkB mediated signaling enhances invasiveness through inducing EMT, and activation of MMP-2 and MMP-9 **(A)** (a) K252a abrogates BDNF-mediated AKT phosphorylation in 3 GBC cell lines. Treatment with K252a abrogates BDNF-mediated MEK phosphorylation in TGBC and TYGBK-1, although addition of rhBDNF or K252a did not affect the MEK phosphorylation status in NOZ. (b) TrkB phosphorylation status in 3 GBC cell lines after incubated with rhBDNF (100 ng/ml) with or without K252a (500 nM) preincubation for 18 h. **(B)** Cell aspect ratio and morphological changes for 48 h after control siRNA or TrkB siRNA transfection. Original magnification is 200×. Scale bar; 100 μm. **(C)** Western blot analysis of E-cadherin, Vimentin, and EMT related transcription factors (Slug, Snail, and Twist) in 3 GBC cell lines for 24 h (a) after treatment with rhBDNF (100 ng/ml) with or without K252a (500 nM) pretreatment, or (b) after transfection with control siRNA, TrkB siRNA, or BDNF siRNA. **(D)** Western blotting and gelatin zymography assay for TrkB siRNA transfected 3 GBC cell lines (a–c). Invasion assay for MMP2 and MMP-9 siRNA transfected 3 GBC cell lines (d–f). *; P<0.05. Bar; SD.

Among the 5 GBC cell lines that we studied, NOZ is known to exhibit K-ras mutation. In TGBC2TKB and TYGBK-1 cells, addition of rhBDNF induced MEK phosphorylation, and K252a treatment abrogated this effect; whereas in NOZ cells, MEK phosphorylation status was unchanged by BDNF or K252a treatment (Figure 4A-a). TrkB phosphorylation status was also examined, as shown in Figure 4A-b. We supposed that this difference results from the K-ras mutation in NOZ cells. We used TGBC2TKB and TYGBK-1 as K-ras wild-type cells, and NOZ as K-ras mutant cells in the following experiments.

EMT is considered to be an important factors involved in cancer invasion [9]. Therefore, we investigated the effect of BDNF/TrkB signaling on EMT. We first observed the morphology of TrkB-inhibited cells by analyzing the cell aspect ratio. In phase-contrast images (Figure 4B), we found that control siRNA-treated cells showed a spindle-like morphology, which is one of the major characteristics of EMT. In contrast, cells transfected with TrkB siRNA were notably rounded, with epithelial morphology. TrkB siRNA transfection substantially reduced the percentage of spindle-shaped cells, and the cells seem to gain cell–cell contacts (Figure 4B).

Second, to determine whether these morphological transformations represent EMT, we analyzed the expression of E-cadherin, vimentin, and several EMT transcription factors (slug, snail, and twist) by western blotting. rhBDNF exposure enhanced the expression of vimentin and EMT transcription factors (slug, snail, and twist), and these increases in expression were inhibited by K252a. The decrease in E-cadherin expression caused by rhBDNF treatment was abrogated by K252a. Conversely, enhanced expression of vimentin in response to rhBDNF treatment was abrogated by K252a (Figure 4C-a). Consistent with the results shown in Figure 4C-a, an increase in the expression of E-cadherin and a decrease in the expression of vimentin and transcription factors (slug, snail, and twist) were observed in western blots of TrkB siRNA- or BDNF siRNA-transfected GBC (Figure 4C-b).

These results demonstrate that BDNF/TrkB signaling induces EMT by upregulating transcription factors in GBC.

### TrkB-mediated signaling-induced invasion is also mediated through MMP-2 and MMP-9

Matrix metalloproteinases (MMPs) are known to be key enzymes involved in degradation of the extracellular matrix [9]. Previously, we showed that MMP-2 and MMP-9 promote GBC invasion [10]. To explore how TrkB-mediated signaling induced invasion in GBC cells, we examined whether MMP-2 and MMP-9 were altered in 3 GBC cell lines. The expression and enzyme activity of MMP-2 and MMP-9 in TrkB siRNA-transfected GBC cells significantly decreased compared with in control siRNA-transfected cells, as measured by western blotting and gelatin zymography (Figure 4D a-c). We then investigated the effect of MMP-2 and MMP-9 knockdown on the invasiveness of GBC cells. As shown in Figure 4D d-f, knockdown of MMP-2 and MMP-9 significantly inhibited the invasiveness of GBC. These results indicate that the enhanced cell invasion induced by TrkB-mediated signaling also occurs via upregulation of the MMP-2 and MMP-9 pathway in GBC.

### TrkB-mediated signaling promotes HIF-1α, VEGF-A, VEGF-C, and VEGF-D expression

**Figure 5:**
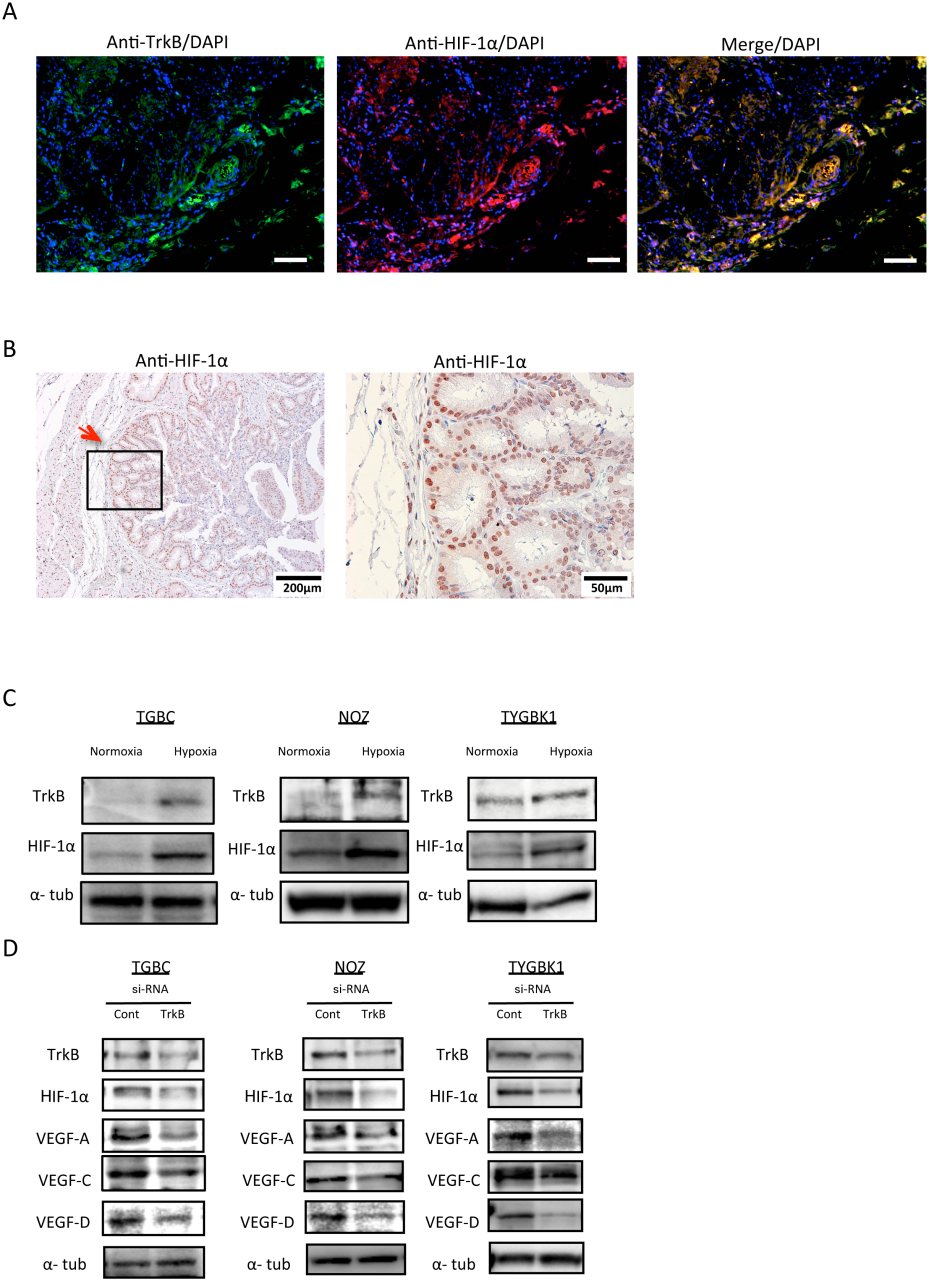
TrkB-mediated signaling promotes HIF-1α, VEGF-A, VEGF-C, and VEGF-D expression **(A)** Representative pictures of immunofluorescence staining of surgically resected gallbladder cancer specimens for TrkB and HIF-1α. Original magnification is 100×. Scale bar; 100 μm. **(B)** Immunohistochemistry staining of surgically resected gallbladder cancer specimens for HIF-1α. Red arrow shows the invasive front. Original magnification is 400×(right) and corresponding areas (boxed areas) with lower magnification 100×(left). **(C)** Western blot analysis of TrkB and HIF-1α expression in 3 GBC cell lines (TGBC, NOZ, and TYGBK-1) under normoxic or hypoxic conditions. **(D)** Western blot analysis of TrkB, HIF-1α, VEGF-A, VEGF-C, and VEGF-D in 3 GBC cell lines transfected with control siRNA or TrkB siRNA.

A previous report indicated a relationship between BDNF/TrkB signaling and levels of vascular endothelial growth factor (VEGF) and hypoxia-inducible factor-1α (HIF-1α) [11]. We therefore investigated the relationship between TrkB-mediated signaling, and VEGF and HIF-1α in GBC. We evaluated TrkB and HIF-1α expression in human GBC specimens by immunofluorescence staining and immunohistochemistry. Interestingly, HIF-1α was highly expressed in the invasive front, where TrkB is also highly expressed (Figure 5A). Furthermore, HIF-1α expression was mainly observed in nuclei (Figure 5B). This result indicates a correlation between TrkB and HIF-1α. Next, to investigate the effect of hypoxia, a cancer microenvironment, we compared TrkB and HIF-1α expression in normoxic and hypoxic conditions. According to western blotting results, TrkB and HIF-1α expression increased under hypoxic conditions (Figure 5C). Finally, we examined whether TrkB knockdown affected HIF-1α, VEGF-A, VEGF-C, or VEGF-D expression. TrkB siRNA transfection significantly inhibited expression of HIF-1α, VEGF-A, VEGF-C, and VEGF-D (Figure 5D). These findings suggest that TrkB-mediated signaling is involved in expression of HIF-1α and VEGFs, especially in the invasive front.

### Inhibition of TrkB suppresses tumorigenicity and tumor proliferation *in vivo*

**Figure 6:**
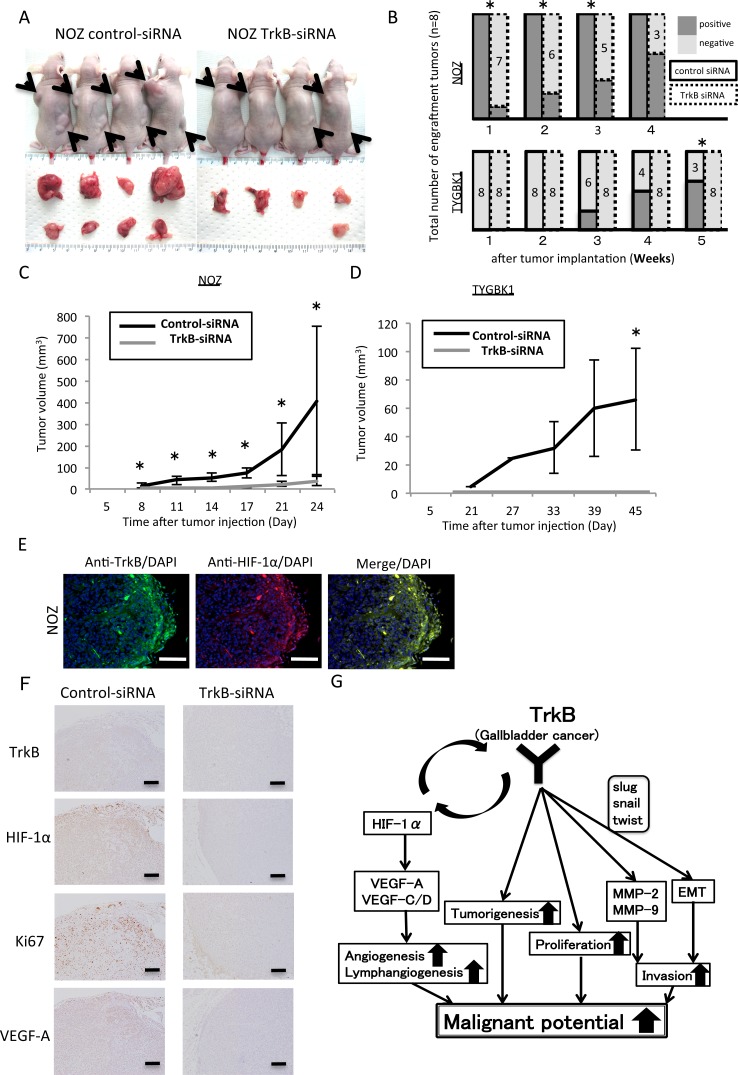
Inhibition of TrkB suppresses tumorigenicity and tumor proliferation *in vivo* **(A)** Representative photograph of NOZ xenograft tumors on day 24. Matrigel mixtures containing 1×10^6^ NOZ cells transfected with control siRNA or TrkB siRNA were implanted subcutaneously in the flank site of BALB/c nude mice (n=4 per group). Black arrows show the each tumors. **(B)** Tumorigenicity of NOZ and TYGBK-1 transfected with control siRNA or TrkB siRNA. **(C, D)** The xenograft tumors volume of control siRNA group (n=4) and TrkB siRNA group (n=4) in NOZ and TYGBK-1. Original magnification is 200×. Scale bar; 100 μm. **(E)** Representative pictures of Immunofluorescence staining of resected xenograft tumors from nude mice for TrkB (green; Alexa 488) and HIF-1α (red; Alexa 594). Original magnification is 200×. Scale bar; 100 μm. **(F)** Immunohistochemistry staining of resected NOZ xenograft tumors from nude mice for TrkB, HIF-1α, Ki67, VEGF-A. Original magnification is 100×. Scale bar; 100 μm. **(G)** Model of our findings in this study. *; P<0.05. Bar; SD.

To determine whether the changes induced by TrkB-mediated signaling observed *in vitro* are reflected *in vivo*, NOZ (K-ras mutant) and TYGBK-1 (K-ras wild-type) cells transfected with control siRNA or TrkB siRNA were subcutaneously injected into both dorsal regions of athymic nude mice (4 mice per group, Figure 6A). Both cell lines transfected with TrkB siRNA demonstrated significantly decreased tumorigenicity (Figure 6B). Interestingly, 7 of the 8 subcutaneous tumors that formed from control siRNA-treated NOZ cells exhibited decreased mobility at 3 weeks, suggesting local deep infiltration. Tumors from mice injected with TrkB siRNA-transfected NOZ and TYGBK-1 cells were significantly smaller than those from mice injected with control siRNA-transfected cells (Figure 6C, 6D). TrkB siRNA-transfected TYGBK-1 cells showed no sign of developing into tumors during the 45-day observation period (Figure 6D). These results suggest that TrkB-mediated signaling plays a pivotal role in tumor proliferation and tumorigenesis *in vivo*. We then conducted immunofluorescence and immunohistochemistry staining in the resected xenograft tumors. Immunofluorescence staining revealed coexpression of TrkB and HIF-1α in the invasive front (Figure 6E). Immunohistochemistry staining indicated TrkB, HIF-1α, Ki67, and VEGF-A expression in the invasive front in the control siRNA-transfected group, and expression of all of these factors was suppressed in the TrkB siRNA-transfected group (Figure 6F).

## DISCUSSION

We confirmed by immunohistochemistry that most GBC tumors overexpressed TrkB (Figure 1A). Furthermore, we revealed that TrkB overexpression in the invasive front correlated significantly with invasion depth, UICC stage (Table 1), and poor patient prognosis (Figure 1D). Xiong L *et al*. reported that BDNF is overexpressed in GBC [12]; another previous report described overexpression of neurotrophins in the invasive front, and demonstrated that higher neurotrophin expression is associated with unfavorable clinicopathological findings and poor patient prognosis [13]. Consistent with our results, Tanaka *et al*. showed that TrkB expression in the invasive front is associated with aggressive tumor phenotype in gastric cancer [14]. We believe that BDNF/TrkB signaling is activated in GBC, specifically in the invasive front (Figure 1B). Furthermore, TrkB was overexpressed at the tumor margin of xenograft tumors. Our results suggest that TrkB expression in the invasive front could be useful as a potential prognostic biomarker for GBC.

Addition of rhBDNF did not affect the proliferation of 5 GBC cell lines (Figure 2A-b). Moreover, BDNF siRNA transfection did not result in a significant difference in proliferation (Figure 2D). There is a possibility that ligands other than BDNF have an effect on the proliferation of GBC cells, such as TGBC2TKB, GBd15, and TYGBK-8. A previous study showed that nerve growth factor (NGF) family members such as NGF, neurotrophin-3, and neurotrophin-4/5, which are other ligands of TrkB, influence cancer proliferation [15]. We found that TrkB siRNA transfection decreased proliferation, indicating that TrkB-mediated signaling is involved in proliferation in GBC. *In vivo*, xenograft tumor volume and Ki67 expression were significantly reduced in the TrkB siRNA-transfected group compared with in the control siRNA-transfected group (Figure 6C, 6D, 6F). These results support our *in vitro* findings. Our results suggest that TrkB-mediated signaling enhanced the proliferation of GBC cells.

Hypoxia, a tumor microenvironment, induces VEGF-A and VEGF-C/−D expression via HIF1α, promoting angiogenesis and lymphangiogenesis respectively [16]. Furthermore, a recent report showed that angiogenesis and lymphangiogenesis promote hematogenous and lymphatic metastasis, resulting in poor prognosis [17]. These findings prompted us to analyze the relationship between hypoxia and TrkB or HIF-1α; we found that expression of these factors was elevated under hypoxic conditions (Figure 5C). Moreover, TrkB siRNA transfection decreased expression of HIF-1α and VEGFs (VEGF-A, VEGF-C, and VEGF-D) (Figure 5D). Nakamura *et al*. previously reported that BDNF/TrkB signaling is required for HIF-1α expression [18]. Conversely, a prior report showed that HIF-1α is a transcription factor of TrkB [19]. This discrepancy is explained by the existence of an HIF-1α-mediated signaling pathway that includes TrkB, and which has an mTOR-mediated autoregulatory loop [20]. We hypothesize that TrkB siRNA transfection would inhibit this autoregulatory loop, resulting in decreased HIF-1α expression. Our immunohistochemistry results indicate a tendency for lymph node metastasis to occur in cases with high TrkB levels (P = 0.1375; Table 1). Co-expression of TrkB and HIF 1α were visualized by immunofluorescence staining, and immunohistochemistry revealed that VEGFs were more highly expressed in the tumor margin than in the tumor center, where TrkB and HIF-1α were also overexpressed. However, vascular and lymphatic vessel density did not differ significantly between animals implanted with TrkB siRNA-transfected cells and those implanted with control siRNA-transfected cells (data not shown). Our results suggest that TrkB-mediated signaling is involved in expression of VEGFs, thereby influencing hematogenous and lymphatic metastasis.

In our experiments, inhibition of TrkB expression suppressed GBC proliferation, invasion, and expression of VEGF-A and VEGF-C/−D, regardless of the presence or absence of K-ras mutation. Based on our results, a Trk inhibitor, which is under phase I/IIA trial, may be an effective treatment for GBC [21]. K-ras mutation status testing has been highlighted for determination of treatment plan in recent years, and molecular target therapies, such as anti-EGFR therapy, have been developed for several solid cancer types, but not for GBC [22]. A previous report showed that K-ras mutations were found in 19%–40% of patients with GBC [23], and a recent report showed that therapies targeting downstream factors of K-ras may be feasible for patients with K-ras mutations [24]. A potential limitation of our experiment is that we were unable to analyze K-ras mutation in clinical GBC specimens or investigate differences in suppression of GBC malignancy resulting from the presence or absence of K-ras mutation *in vitro*.

Figure 6G depicts an overall schematic of our present findings. In summary, TrkB-mediated signaling increase proliferation, tumorigenesis, and invasiveness by promoting EMT and activation of MMP-2/MMP-9. Moreover, TrkB-mediated signaling is implicated in the expression of HIF-1α and VEGF-A/−C/−D, which promote angiogenesis and lymphangiogenesis. Inhibition of the TrkB-mediated signaling pathway may lead to suppression of proliferation, invasiveness, angiogenesis, lymphangiogenesis, and tumorigenesis. Taken together, we showed the biological significance of BDNF/TrkB signaling in GBC for the first time. Our findings indicate that TrkB-mediated signaling is involved in the induction of malignant phenotype in GBC, and could be an attractive therapeutic target for GBC.

## MATERIALS AND METHODS

### Cell lines

We used 5 GBC cell lines: TGBC2TKB, NOZ [25], GBd15 [26], TYGBK-1 [27], and TYGBK-8. TGBC2TKB was purchased from Riken Cell Bank (Tsukuba, Japan). NOZ, TYGBK-1, and TYGBK-8 were purchased from the Japanese Collection of Research Bioresources (JCRB) bank. All cell lines were cultured according to the supplier's specifications. There was no mycoplasma contamination in any of the cell lines with mycoplasma detection kit (Lonza, Basel, Switzerland). For normoxic conditions, cells were cultured in 5% CO_2_ and 95% air and for hypoxic conditions, cells were cultured in 1% O_2_, 5% CO_2_, and 94% N_2_, in a multigas incubator (Sanyo, Tokyo, Japan).

### Cell proliferation assay

All GBC cell lines were seeded onto 96-well plates at 5000 cells/well and incubated with or without recombinant human BDNF (Peprotech, Rocky Hill, NJ, USA) or the Trk inhibitor K252a (Alomone Labs, Jerusalem, Israel) for 24, 48, or 72 h. Cell proliferation was assessed by absorbance (Biotrak visible plate reader, Amersham Biosciences) at 492 nm (reference wavelength 620 nm) using Cell Count Reagent SF (Nacalai Tesque, Kyoto, Japan). Forty-eight hours after TrkB small interfering RNA (siRNA) and BDNF siRNA transfection, the cells were reseeded onto 96-well plates and proliferation rate was measured.

### Cell invasion assay

The invasiveness of the GBC cell lines was assessed by Matrigel invasion assay as described previously [10]. Briefly, cells (2 × 10^5^) were placed in the upper chamber with or without recombinant human BDNF or the Trk inhibitor K252a, and subsequently incubated for 18 h. Alternatively, cells were treated for 48 h with TrkB siRNA and/or BDNF siRNA, and placed in the upper chamber and incubated for 18 h. The cells that migrated to the lower side of the filter were fixed and stained with Diff-Quik reagent (Sysmex, Kobe, Japan) and then counted under a light microscope (Nikon Eclipse TE 300, Nikon, Tokyo, Japan).

### RNA interference

ON-TARGETplus™SMARTpool siRNA targeting TrkB (L-003160), BDNF (L-017626), MMP-2 (L-005959), and MMP-9 (L-005970), and negative control siRNA (ON-TARGETplus™Control non-targeting siRNA, D-001810) were purchased from Dharmacon (Lafayette, CO, USA). Following overnight attachment to a 6-well plate, 3 × 10^5^ cells per well were transfected for 48 hours using Lipofectamine RNAiMAX (Invitrogen, Carlsbad, CA, USA) according to the manufacture's protocol.

### Western blot analysis

Western blotting was performed as described previously [28]. The protein-transferred membranes were incubated overnight at 4°C with primary antibodies for TrkB (1:200, sc-8316, Santa Cruz Biotechnology, Santa Cruz, CA, USA), p-TrkB (1:500, ab197072, Cambridge, MA, USA), BDNF (1:200, sc-546, Santa Cruz Biotechnology), MMP-2 (1:200, sc-10736, Santa Cruz Biotechnology), MMP-9 (1:200, sc-6840, Santa Cruz Biotechnology), E-cadherin (1:200, sc-7870, Santa Cruz Biotechnology), vimentin (1:200, sc-6260, Santa Cruz Biotechnology), SLUG (1:200, sc-15391, Santa Cruz Biotechnology), SNAIL (1:200, sc-10433, Santa Cruz Biotechnology), twist (1:200, sc-15393, Santa Cruz Biotechnology), VEGF-C (1:200, sc-7133, Santa Cruz Biotechnology), VEGF-D (1:200, sc-7603, Santa Cruz Biotechnology), p-MEK-1/2 (1:200, sc-7995, Santa Cruz Biotechnology), Erk1/2 (1:200, No9102 Cell signaling Technology), p-Akt1/2/3 (1:200, sc-101629, Santa Cruz Biotechnology), or Akt1/2/3 (1:200, sc-8312, Santa Cruz Biotechnology). Peroxidase-linked secondary antibodies (Amersham Biosciences, Piscataway, NJ, USA) were subsequently added and the membranes were further incubated for 1 h at room temperature. The antibodies for α-tubulin (1:1000, Sigma-Aldich, St. Louis, MO, USA) and β-actin (1:200, sc-47778, Santa Cruz Biotechnology) were used as protein loading controls.

### Gelatin zymography

MMP-2 and MMP-9 enzyme activity was assessed by gelatin zymography as described previously [10]. Briefly, siRNA-TrkB was transfected into cells. After 48 h culture, protein samples were extracted and analyzed using a gelatin zymography kit (Cosmobio, Tokyo, Japan) according to the manufacturer's instructions.

### Immunohistochemistry

Tissue samples were obtained from patients with GBC who underwent resection at the Department of Surgery and Oncology, Kyushu University Hospitals, Fukuoka, Japan between 2001 and 2012. Approval for the use of tissues was obtained from patients in accordance with the Ethical Committees for Clinical Study at Kyushu University. Immunohistochemical staining was performed using 4-μm-thick formalin-fixed, paraffin-embedded tissue sections and primary antibodies for TrkB (1:50, sc-8316, Santa Cruz Biotechnology), MIB-1 (1:100, Dako), VEGF (1:100, sc-152, Santa Cruz Biotechnology), or HIF-1α (1:100, sc-8711, Santa Cruz Biotechnology). Endogenous peroxidase activity was blocked for 30 minutes using methanol containing 0.3% hydrogen peroxidase. Sections were incubated with primary antibodies overnight at 4°C, followed by incubation for 40 minutes with the secondary antibodies at room temperature. The reaction products were visualized using diaminobenzidine (DAB). The TrkB staining intensity of each slide was separately scored for the tumor invasion front and the tumor center as follows: 1 researcher and 1 pathologist evaluated the whole section on a blind basis and scored the staining intensity as none (0), weak (1), moderate (2), or strong (3) (Figure 1B 1a–1d).

### Fluorescence immunohistochemistry

Slides with xenograft tumors and GBC cells from patients were immunostained with primary antibodies for TrkB (1:100, sc-8316, Santa Cruz Biotechnology) and HIF-1α (1:100, sc-8711, Santa Cruz Biotechnology), followed by Alexa Fluor 488-labeled and Alexa Fluor 594-labeled secondary antibodies (1:1000, Invitrogen), as described previously [28].

### *In vivo* xenograft tumor model

Five-week-old female athymic nude mice (BALB/c nu/nu) were purchased from Charles River Laboratories Japan (Kanagawa, Japan) and acclimated for 2 weeks. All experimental procedures were approved by the Animal Care and Use Committee of Kyushu University (permit number: A27-324-0). All mice were housed and maintained in a specific pathogen-free animal facility at Kyushu University, and all efforts were made to minimize the number of animals used and their suffering. All experiments were performed in strict accordance with the Guidelines for Proper Conduct of Animal Experiments (Science Council of Japan). NOZ and TYGBK-1 cells transfected with TrkB siRNA or control siRNA were subcutaneously implanted into the flank of nude mice (1 × 10^6^ cells in Matrigel per mouse; n=4 in each treatment group). Tumor size was measured twice a week, and volume was calculated as follows: A × B^2^× 0.5, where A is the longest diameter and B is the smaller of the two perpendicular diameters of the tumor. The mice were euthanized when the tumor size reached 2 cm in diameter or if the animals became moribund during the observation period.

### Statistical analysis

All data are represented as mean ± standard deviation (SD). A χ^2^ test was used to analyze tumorigenicity in mice, and the relationship between clinicopathological parameters in patients. The Student's t-test was used for comparison of mean values between two groups. Survival curves were plotted using the Kaplan–Meier method and analyzed using two-sided log-rank tests. A value of P < 0.05 was considered significant.

## SUPPLEMENTARY MATERIALS FIGURES



## Abbreviations

BTC: bile tract cancer
GBC: gallbladder cancer
BDNF: brain-derived neurotrophic factor
Trk: tropomyosin-related kinase
rhBDNF: recombinant human BDNF
siRNA: small interfering RNA
EMT: epithelial-mesenchymal transition
MMP: Matrix metalloproteinase
HIF-1α: hypoxia-inducible factor-1α
VEGF: vascular endothelial growth factor
SD: standard deviations
