# Supplementary material for: Tropomyosin-related kinase B mediated signaling contributes to the induction of malignant phenotype of gallbladder cancer
Source: Oncotarget. 2017 Mar 10;8(22):36211–24. doi: 10.18632/oncotarget.16063 (PMC5482650; doi:10.18632/oncotarget.16063)
Supplement: Supplementary file 1 [file oncotarget-08-36211-s001.pdf]

Tropomyosin-related kinase B mediated signaling contributes to the induction of malignant phenotype of gallbladder cancer

SUPPLEMENTARY FIGURES

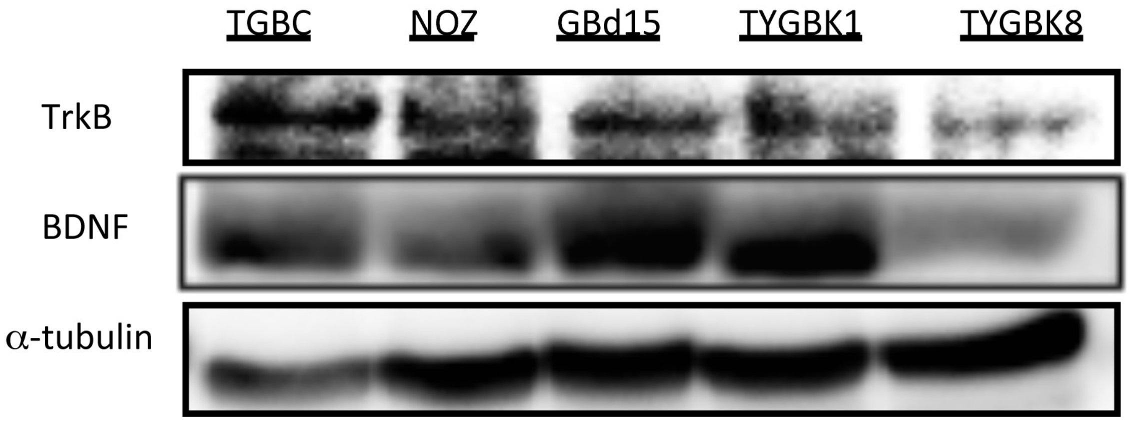

Supplementary Figure 1: Western blot analysis of TrkB and BDNF in 5 GBC cell lines.

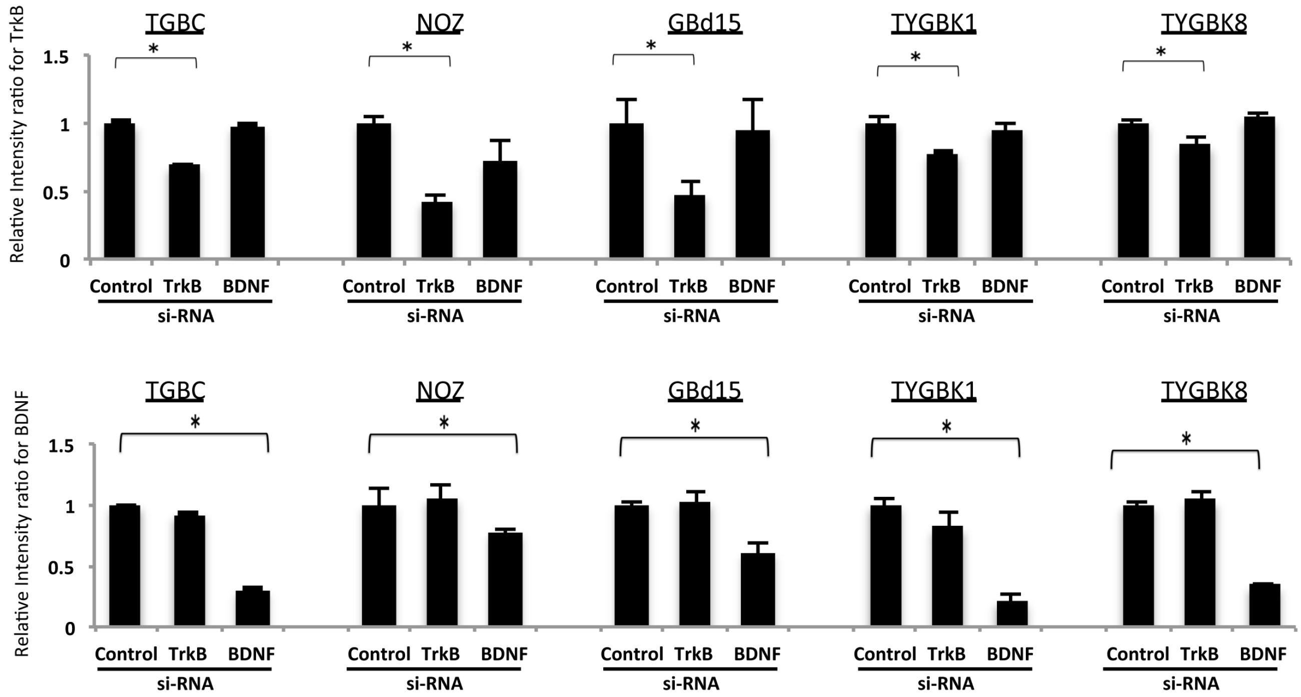

Supplementary Figure 2: Relative intensity ration of TrkB and BDNF in 5 GBC cell lines transfected with TrkB siRNA or BDNF siRNA. Scanned images of the respective immunoblot (Figure 2C) were calculated using NIH ImageJ (Scion, Frederick, MD, USA) in the linear range and adjusted based on the respective α-tubulin intensity.

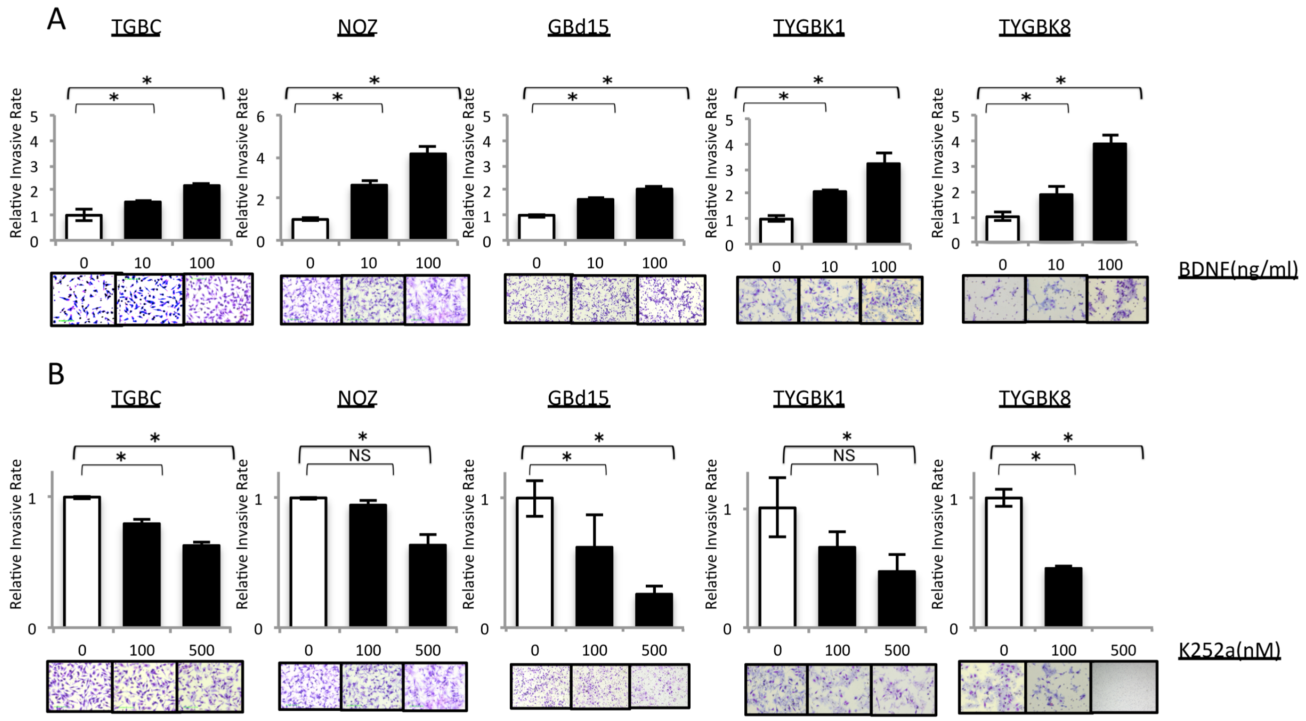

Supplementary Figure 3: (A) Invasion assay of 5 GBC cell lines incubated for 18 h with rhBDNF (at 0, 10, or 100 ng/ml) as indicated. **(B)** Invasion assay of 5 GBC cell lines incubated for 18 h with K252a (at 0, 100, or 500 nM). *; P<0.05. Bar; SD. NS; not significant. Original magnification is 100×.
